# Evolutionary History of the Adult β‐Globin Gene in Wild Sheep: First Sequences From the Argali (*Ovis ammon*) and the Urial (*Ovis vignei*)

**DOI:** 10.1002/ece3.73031

**Published:** 2026-02-23

**Authors:** Paolo Mereu, Chiara Multineddu, Daria Sanna, Marco Zedda, Antonio J. Lepedda, Giovanni G. Leoni, Monica Pirastru

**Affiliations:** ^1^ Dipartimento di Scienze Biomediche Università di Sassari Sassari Italy; ^2^ Dipartimento di Medicina Veterinaria Università di Sassari Sassari Italy

**Keywords:** argali, HBBA, HBBB, molecular dating, Ovis, phylogeny, Urial

## Abstract

The β‐globin gene cluster is a key model for studying molecular adaptation and gene family evolution in vertebrates, exhibiting dynamic duplication and deletion events in ruminants, particularly within the subfamily Caprinae. However, the complete nucleotide sequences of the adult β‐globin gene remain unknown for several ecologically important wild sheep species, such as the Argali and the Urial, hindering robust phylogenetic and adaptive studies. Genomic DNA was extracted from wild Argali and Urial samples, and the full coding region of the adult β‐globin gene was sequenced and compared against a comprehensive dataset of Bovidae and Cervidae. Sequence comparison confirmed high divergence between paralogous genes (HBBA and HBBB), and critically, revealed that Argali hosts two distinct adult β‐globin haplotypes, one harboring a HBBA gene and the other a HBBB gene. Phylogenetic analysis revealed that sequences clustered consistently by paralog type (HBBA vs. HBBB) with both the Caprinae and Bovinae lineages. This pattern suggests independent evolutionary trajectories for each paralog, likely driven by concerted evolution. Molecular dating estimated the duplication event leading to the HBBA/HBBB paralogs in Caprinae at approximately 2.55 million years ago, supporting a rapid, recent diversification of the β‐globin cluster in *Ovis* during the Pleistocene. Our analysis provides the newly characterized sequences of the Argali and Urial adult β‐globin gene and detected ancestral polymorphism in Argali. The divergence time estimates and the clustering by paralog type provide a valuable temporal and evolutionary framework for understanding the complex dynamics of multigene families and offer a foundation for future studies investigating potential high‐altitude adaptive selection in wild *Ovis* species.

## Graphical abstract

This study addresses a key gap in the understanding of molecular adaptation by providing the first complete adult β‐globin gene sequences for two ecologically significant wild sheep species, the Argali ( *Ovis ammon* ) and the Urial ( *Ovis vignei* ).

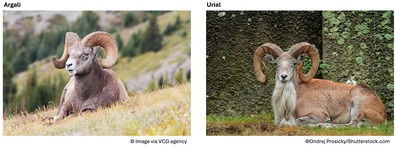

## 1 Introduction

The β‐globin gene family is known to be an effective model for studying molecular adaptation, gene regulation, and phylogenetic relationship in vertebrates (Storz 2018). Within ruminants (family Bovidae), its evolution has been particularly dynamic, characterized by recurrent, large‐scale duplication and deletion events followed by divergence and specialization that have resulted in striking lineage‐specific genomic architectures. Ruminants show classical hemoglobin (Hb) switching: The multiple copies of β‐globin genes are expressed at different developmental stages—embryonic, fetal, and adult—each with distinct functional properties tailored to the organism's oxygen requirements. While the bovine lineage (subfamily Bovinae) retains the ancestral ruminant pattern of a single adult β‐globin gene and a fused, pan‐specific ε/β‐like gene (MacEachern et al. 2009), the Caprinae subfamily lineage underwent a subsequent duplication event. This gave rise to a distinct genomic arrangement of the β‐globin gene cluster, named A‐haplotype, organized as a triplicated four‐gene set, totaling 12 genes, and is distinguished by the presence of a third β‐globin paralog, the preadult β^C^ (Rangan et al. 2021). Notably, the juvenile/preadult β^C^ and the adult‐expressed β^A^ globins display different oxygen‐affinity properties and can be differentially regulated, providing a refined mechanism for physiological adaptation. Adult sheep and goats exhibit well‐documented switching and some unusual regulatory dynamics, such as the reactivation of the preadult β^C^ gene under certain stress or pathological conditions (Pirastru et al. 2009).

The presence of this additional β^C^ gene block marks a critical point of divergence between Bovinae and Caprinae lineages. This triplicated arrangement has been described in goats ( *Capra hircus* Linnaeus, 1758), aoudad ( *Ammotragus lervia* Pallas, 1777), and in two *Ovis* species including Sardinian mouflon (*Ovis gmelini musimon* Pallas, 1811) and a subset of domestic sheep ( *Ovis aries* Linnaeus, 1758), known as the A‐haplotype sheep (Manca et al. 2006; Pirastru et al. 2009). The fact that this identical triplicated architecture is shared among these species provides strong evidence for a common ancestral state. Therefore, this triplicated locus was probably present in a common ancestor of Caprinae after its divergence from Bovinae.

The genus *Ovis* represents a fascinating group for evolutionary studies, having radiated across a wide array of ecological niches, from lowland deserts to high‐altitude mountain plateaus. This ecological diversity is reflected in their β‐globin gene cluster that, in addition to the triplicate arrangement above described, can also host a duplicated four‐gene set (B‐haplotype) harboring the adult β^B^ gene. This arrangement lacking the β^C^ gene is observed in a different group of domestic sheep (B‐haplotype sheep), Cyprian mouflon (*O. gmelini ophion* Blyth, 1841), Bighorn ( *Ovis canadensis* Shaw, 1804), and Snow sheep ( *Ovis dalli* Nelson, 1884) (Manca et al. 2006). Although the B‐haplotype sheep locus could bear a striking resemblance to the bovine locus, this similarity does not stem from a direct, recent shared ancestry. Indeed, the bovine duplicated locus arose from an ancestral duplication event that occurred before the divergence of cows and goats, estimated to be more than 18–20 million years ago (Jiang et al. 2015). Conversely, the B‐haplotype in sheep is believed to have resulted from a much more recent deletion from a triplicated locus. A detailed resequencing study of the B‐haplotype suggests it is at least 2–3 million years old, still a far more recent event than the deep evolutionary split between Bovinae and Caprinae. This suggests the shared duplicated state in cattle and B‐haplotype sheep as a case of convergent evolution or secondary loss, rather than retention of a shared ancient trait (Jiang et al. 2015). The different evolutionary timelines and mechanisms leading to the duplicated state highlight the distinct evolutionary pathways within these subfamilies. The complex relationships between these gene clusters are summarized in Table 1.

**TABLE 1:** Comparative genomic organization of the β‐globin gene cluster in Bovidae.

Species	Locus structure	Genes	Key evolutionary event
Cow	Duplicated	ϵ, ψβ, β^A^, β^F^	Ancestral duplication before Caprinae divergence
Goat	Triplicated	ϵ, ψβ, β^C^, β^A^, β^F^	Assumed ancestral state for Caprinae
A‐Haplotype sheep	Triplicated	ϵ, ψβ, β^C^, β^A^, β^F^	Retention of ancestral Caprinae locus
B‐Haplotype sheep	Duplicated	ϵ, ψβ, β^B^, β^F^	Recent deletion from a triplicated locus
Tibetan antelope	Duplicated	ϵ, ψβ, β^C^, β^F^	Secondary deletion from a triplicated locus

Protein‐coding genes like β‐globin genes, which are under selective pressure and exhibit a workable rate of evolution, are excellent candidates for shedding light on intricate phylogenetic relationships (Hardison 2012; Storz 2016). Their sequences can reveal not only phylogenetic signals but also potential signatures of positive selection related to environmental adaptation.

**FIGURE 1:**
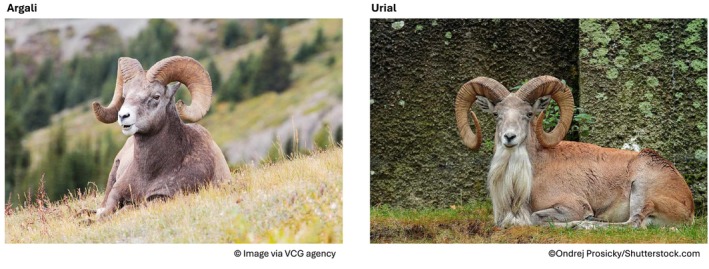
Argali and Urial wild sheep. The pictures are freely available on the internet under Creative Commons License.

Despite their utility, the complete nucleotide sequences of the adult β‐globin gene from some wild *Ovis* species remain entirely unknown. The Argali ( *Ovis ammon* Linnaeus, 1758) and the Urial ( *Ovis vignei* Blyth, 1841) are two phylogenetically distinct and ecologically critical wild sheep species (Figure 1). The Argali thrives in the high‐altitude environments of Central Asia, while the Urial occupies a range of intermediate elevations across South and Central Asia. Their evolutionary relationships with each other and to domestic sheep are still refined using primarily mitochondrial DNA and neutral markers (Chen et al. 2021; Dotsev et al. 2023; Lv et al. 2024; Mereu et al. 2024).

The absence of β‐globin gene sequences from these species represents a significant gap in our understanding. It prevents a robust phylogenetic analysis of a functional nuclear marker within *Ovis* and, more importantly, hinders investigations into the molecular basis of potential adaptive evolution.

Therefore, the objectives of this study were to: (i) isolate and sequence the complete coding regions of the adult β‐globin genes from the Argali and the Urial; (ii) characterize these sequences by comparing them to those of domestic sheep and other bovids to identify polymorphisms and potential functionally important substitutions; and (iii) perform a phylogenetic analysis to elucidate the evolutionary history of the β‐globin genes within the Caprinae subfamily. This study provides the first genetic data for this critically important gene family in Argali and Urial, establishing a foundation for future research into the molecular physiology of adaptation and the evolutionary dynamics of multigene families in wild species.

## 2 Material and Methods

### 2.1 Sample Collection

The biological samples (hairs) were obtained from one Urial (Turkmenistan), one Bighorn (Canada), and two Argali (Kazakhstan, Kara‐Tau mountain) individuals. This study adhered to ethical standards for animal research, which prioritize animal well‐being. All the animal procedures were performed in strict accordance with the guidelines of the Ethics Committee of Sassari University, Italy, which also approved this study.

### 2.2 DNA Extraction, Amplification, and Sequencing

Genomic DNA was extracted from hair using the NucleoSpin Tissue XS kit (Macherey‐Nagel, Germany), according to the manufacturer's protocol. DNA quality and concentration were assessed via spectrophotometry using a ND‐8000 (NanoDrop Technologies, Thermo Fisher Scientific Inc., Wilmington, DE, USA).

**FIGURE 2:**
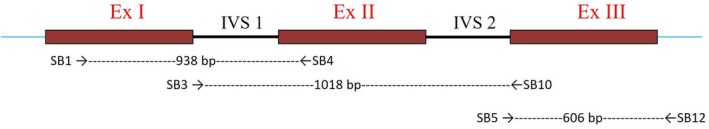
PCR amplification strategy based on the use of three primer pairs to isolate the adult β‐globin gene sequences.

Oligonucleotide primers for the polymerase chain reaction (PCR) amplification were designed on the basis of sheep *HBBA* and *HBBB* sequences available on GenBank under the accession numbers DQ352470‐1 (Manca et al. 2006; Pirastru et al. 2003). A 938 bp fragment, from the promoter region to the second exon, was amplified using SB1 (5′‐AATAATCCATCCACATAGTCTTGAA‐3′, from positions −392 to −368 with respect to the Cap site) and SB4 (5′‐CCTCTTCTCCATTCTAAACTGT‐3′, from +572 to +593) primers. A 1018 bp fragment, from the second to the third exon, was amplified using SB3 (5′‐TGACTTCCTCTGACCTTGT‐3 from +229 to +247) and SB10 (GTCCACGGATTTCTCCAGGC‐3′ from +1273 to +1292) primers. The remaining portion of the gene was obtained using SB5 (5′‐TATTCTTGTGCTTCCCTTGTGG‐3′ from +1144 to +1165) and SB12 (5′‐TACAACCATCTTGTCATTGGG‐3′ from +1772 to +1792) primers (Figure 2). PCR products were purified by electrophoresis on a 1.2% agarose gel using the Montage DNA Gel extraction kit (Millipore, Burlington, MA, USA) and then sequenced by means of automated capillary sequencing using the PCR primers. Nucleotide sequencing was outsourced to Macrogen Europe, with each read performed in duplicate. This redundancy was implemented to maximize the precision of base calling and to mitigate potential sequencing errors. Processed sequences were visualized using FinchTV 1.4.0 (Geospiza Inc.) and assembled into contigs, after identifying overlapping areas on Clustal X 2 (Larkin et al. 2007).

### 2.3 Homologous Sequences Retrieving and Analyses

BLAST queries with both the Argali and the Urial sequences were carried out against the GenBank database, and the dataset was complemented by adding 26 homologous sequences from Bovidae and three from Cervidae species. A further sequence from wild boar was downloaded and used as outgroup (Table 2).

**TABLE 2:** β‐globin gene sequences included in the dataset.

GenBank #	Species	Common name	Haplotype	Sampling location
This study	*Ovis ammon* (1)	Argali	HBBB	Kazakhstan, Kara‐Tau mountain
	*Ovis ammon* (2)		HBBA	Kazakhstan, Kara‐Tau mountain
	*Ovis canadensis*	Bighorn	HBBB	Canada, Rocky mountain
	*Ovis vignei*	Urial	HBBA	Turkmenistan
DQ352472	*Ammotragus lervia*	Barbary sheep	HBBA	Egypt
DQ350619	*Capra hircus*	Goat	HBBA	Italy, Sardinia
M15387	*Capra hircus*		HBBA(Ala)	n.a.
DQ352470	*Ovis aries*	Domestic sheep	HBBA	Italy, Sardinia
DQ352471	*Ovis aries*		HBBB	Italy, Sardinia
ON586155	*Ovis aries*		HBBB	Brazil
X14727	*Ovis aries*		HBBB	Garner & Lingrel (GL)
DQ366843	*Ovis aries*		HBBK	Chios
DQ352468	*Ovis gmelini musimon*	Sardinian mouflon	HBBA	Italy, Sardinia
DQ352469	*Ovis gmelini ophion*	Cyprian mouflon	HBBB	Cyprus
AM886147	*Bubalus bubalis*	Water buffalo	HBB(A)	Italy, Campania
AM886148	*Bubalus bubalis*		HBB(T)	Italy, Campania
AB512666	*Bos grunniens*	Yak	HBB‐4Ha	Bhutan
AB512664	*Bos grunniens*		HBBA	Bhutan
AB512630	*Bos indicus*	Zebu	HBBA	Vietnam
AB512636	*Bos indicus*		HBBA	Vietnam
AB512627	*Bos indicus*		HBBB	Vietnam
AB512645	*Bos indicus*		HBB‐X1	Vietnam
AB512641	*Bos indicus*		HBB‐X2‐I	Vietnam
AB512643	*Bos indicus*		HBB‐X2‐I	Laos
AB512629	*Bos indicus*		HBB‐X2‐II	Vietnam
AB512649	*Bos javanicus*	Banteng	HBBB	Bali
AB512647	*Bos javanicus*		HBBX	Bali
AB512624	*Bos taurus*	Cattle	HBBA	Japan
AB512624	*Bos taurus*		HBBB	Japan
KY800429	*Alces alces*	Elk	HBB	n.a.
KY800438	*Cervus elaphus*	Red deer	HBB	n.a.
KY800433	*Dama dama*	Fallow deer	HBB	n.a.
X86791	*Sus scrofa*	Wild boar	HBB	n.a.

Sequences were aligned using the Muscle algorithm implemented in MEGA v. 11 (Tamura et al. 2021). To check for selective pressure on the HBB gene, we estimated the numbers of synonymous and nonsynonymous nucleotide substitutions per site and the *K*_a_/*K*_s_ ratios using the counting method by Nei and Gojobori (1986) implemented in DnaSP v.6 (Rozas et al. 2017).

Both the Bayesian Inference (BI) and Maximum Likelihood (ML) trees were inferred on the nucleotide sequences, using the software MrBayes v. 3.2.4 (Ronquist et al. 2012) and RAxML v. 8 (Stamatakis 2014), respectively, with 10,000 bootstrap replicates. The choice of the best model of nucleotide substitution, the GTR + G + I, was performed with MEGA v.11 on the basis of the BIC and AICc criterion values.

Molecular dating of the main splitting events within Bovidae was inferred using a Bayesian approach as implemented in BEAST v.1.10.4 (Suchard et al. 2018). Overall, a total of four calibration points (CPs) based on fossil records were used: three providing time estimates for nodes within Bovidae and one marking the divergence between Cervidae and Bovidae (Kumar et al. 2017) (Table 3).

**TABLE 3:** Calibration points (CP) used to infer molecular dating.

CP	Divergence (MYA)			Substitution rate	
	Median	Adjusted time	C.I.	Mean + SD	Gamma
*A. lervia/O. canadensis*	6	8.4	5.3–9.4	0.0011 ± 0.0022	0.1214
*B. bubalis/B. indicus*	13.1	—	0.8–14.2	0.0006 ± 0.0019	0.1000
*B. bubalis/O. ammon*	24.6	21.6	19.1–30.8	0.0011 ± 0.0041	0.2533
*D. dama/B. indicus*	22.8	23.7	18.5–27.8	0.0012 ± 0.0042	0.3753

Sequences were analyzed under the GTR + G + I model of sequence evolution, assuming 4 gamma categories, a Yule process speciation rate, and empirical base frequencies. We used a lognormal relaxed clock with uncorrelated rates (ULRC) approach. Two independent Markov chain Monte Carlo runs were carried out with 200,000,000 iterations each and sampled every 10,000 steps after a 10% burn‐in. The runs were combined after checking for convergence > 100. A maximum clade credibility (MCC) tree was generated using *TreeAnnotator 1.10.4* and visualized with *FigTree 1.4.4* (http://tree.bio.ed.ac.uk/).

## 3 Results

Four adult β‐globin gene sequences from three wild *Ovis* species—Argali, Urial and Bighorn –were produced in the present study and deposited in GenBank under the accession number PX441602‐5. The four sequences were quite similar in length, ranging from about 1820 to 1920 bp, and included the entire coding region plus partial 5′ and 3′ region ends. The two Argali sequences were 1828 bp and 1860 bp, respectively, the Urial 1917 bp and the Bighorn 1821 bp. Slight differences were detected at the level of intronic region I (IVS‐1), which in the Bighorn was 126 bp while in the other species 128 bp. More marked were those detected in intron II (IVS‐2), which was 902 bp long in the Urial and one of the two Argali, 900 bp in the Bighorn and 891 bp in the other Argali. The average nucleotide composition was 29.6 T, 21.3 C, 24.7 A and 24.3 G. The dataset was completed by downloading 30 additional homologous sequences from GenBank, for a total of 33 sequences.

### 3.1 Sequence Comparison, Genetic Distances Estimations and Selective Pressure

To quantify the genetic divergence between the newly sequenced β‐globin genes, we calculated pairwise genetic distances within *Ovis* under the Tamura 3‐parameter (T92) model of nucleotide evolution, assuming a rate variation among sites modeled by a gamma distribution (*G* = 1.02) (Table S1).

The greatest genetic distances were observed between the two paralogous genes, *HBBA* and *HBBB*, with values ranging from 0.01334 to 0.02410. The highest divergence was detected between the Sardinian mouflon/A‐haplotype sheep and the B‐haplotype sheep (0.02410 ± 0.00415). Within the same species, the two Argali sequences exhibited a distance of 0.01334 ± 0.00315, with one sequence found more similar to the *HBBA* sheep gene (0.0627 ± 0.00210) and the other to B‐haplotype sheep. Such evidence allowed us to infer that Argali species host two distinct arrangements of the β‐globin gene cluster, as previously described for domestic sheep.

The comparison between sequences of the same paralog (orthologs) showed a higher level of similarity across species. The *HBBB* gene showed low sequence divergence ranging from 0.00000 (*HBBB* Sheep Sar vs. *HBBB* Sheep Bra) to 0.00417 (*HBBB* Argali vs. Bighorn). Similarly, the *HBBA* gene showed a divergence ranging from 0.00277 (*HBBA* Argali vs. Urial) to 0.00487 (*HBBA* Argali vs. Sardinian mouflon).

The counting of synonymous and nonsynonymous substitutions is reported in Table 4. The *ω* = *K*_a_/*K*_s_ ratios were < 1 in all the pairwise sequence comparisons, ranging between 0 and 0.95.

**TABLE 4:** Synonymous and nonsynonymous substitution patterns between domestic sheep and the newly characterized wild species.

Seq 1	Seq 2	SynSub	SynPos	*K*_s_	NSynSub	NSynPos	*K*_a_	*ω*
Argali_HBBB	Argali_HBBA	0.00	106.00	0.0000	2.00	332.00	0.0060	0
Argali_HBBB	Bighorn	0.00	106.00	0.0000	1.00	332.00	0.0030	0
Argali_HBBB	Urial	0.00	106.00	0.0000	2.00	332.00	0.0060	0
Argali_HBBB	HBBB_Sheep	2.00	105.42	0.0192	4.00	332.58	0.0121	0.63
Argali_HBBB	HBBA_Sheep	3.00	106.50	0.0287	4.00	331.50	0.0122	0.42
Argali_HBBA	Bighorn	0.00	106.00	0.0000	1.00	332.00	0.0030	0
Argali_HBBA	Urial	0.00	106.00	0.0000	0.00	332.00	0.0000	0
Argali_HBBA	HBBB_Sheep	2.00	105.42	0.0192	6.00	332.58	0.0183	0.95
Argali_HBBA	HBBA_Sheep	3.00	106.50	0.0287	2.00	331.50	0.0061	0.21
Bighorn	Urial	0.00	106.00	0.0000	1.00	332.00	0.0030	0
Bighorn	HBBB_Sheep	2.00	105.42	0.0192	5.00	332.58	0.0152	0.79
Bighorn	HBBA_Sheep	3.00	106.50	0.0287	3.00	331.50	0.0091	0.32
Urial	HBBB_Sheep	2.00	105.42	0.0192	6.00	332.58	0.0183	0.95
Urial	HBBA_Sheep	3.00	106.50	0.0287	2.00	331.50	0.0061	0.21
HBBB_Sheep	HBBA_Sheep	3.00	105.92	0.0289	8.00	332.08	0.0245	0.85

Abbreviations: *ω* = K_a_/K_s_ ratio, *K*_a_ = Nonsynonymous rate (NSynSub/NSynPos ratio), *K*_s_ = Synonymous rate (SynSub/SynPos ratio), NSynPos = number of nonsynonymous sites, NSynSub = number of non‐synonymous substitutions, SynPos = number of synonymous sites, SynSub = number of synonymous substitutions.

### 3.2 Phylogenetic Analysis

**FIGURE 3:**
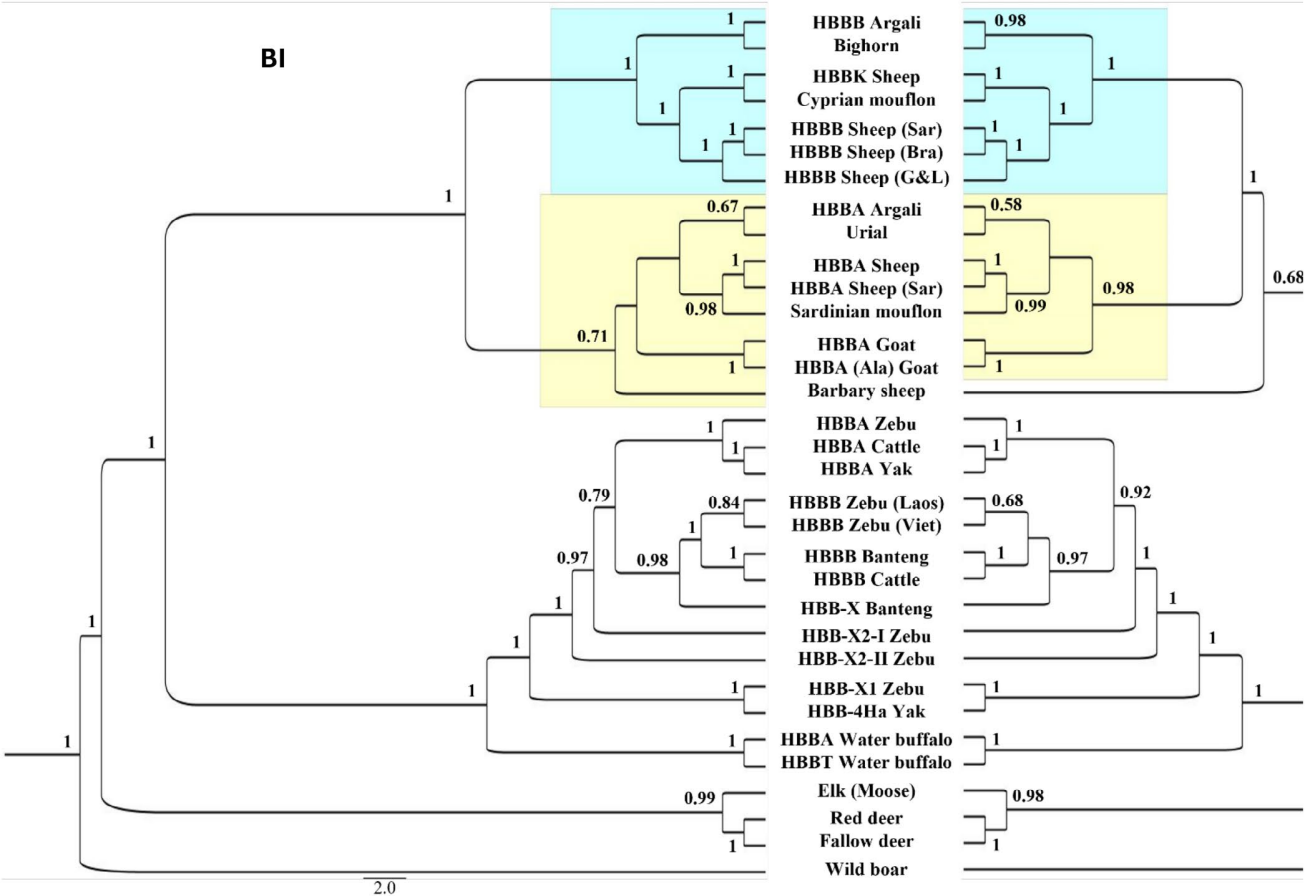
Evolution of the adult β‐globin gene from ruminant species based on the Bayesian Inference (BI; top left) and Maximum Likelihood (ML; top right) methods of tree reconstruction under the GTR model of nucleotide substitution. Nonuniformity of evolutionary rates among sites was modeled by using a discrete Gamma distribution (+G) with 5 rate categories and by assuming that a certain fraction of sites were evolutionarily invariable (+I). Within Caprinae, HBBA and HBBB clades were highlighted in yellow and blue, respectively.

The BI and the ML trees showed the same topology, with the only exception of the Barbary sheep sequence, that was basal to all the Caprinae in the ML tree while clustering within *HBBA* clade in the Bayesian tree (Figure 3). The two phylogenetic trees were both completely resolved and with high statistical support to most of the nodes retrieved (PP for Bayesian tree and bootstrap values for ML tree), the only uncertainty being represented by the position of the Barbary sheep sequence. Within Caprinae group, sequences were split into two clades according to the β‐globin gene cluster haplotypes. One clade, highlighted in blue, included all the sequences from *HBBB* and *HBBK* sheep along with three wild species: the Cyprian mouflon, the Bighorn and the *HBBB* Argali (Figure 3). The first split in this clade separated the Bighorn and *HBBB* Argali from the other sequences, followed by the divergence of *HBBK* sheep and Cyprian mouflon from domestic sheep. The second clade (yellow) was composed of all sequences from goat and *HBBA* domestic sheep, along with three wild species: the Sardinian mouflon, the Urial and the *HBBB* Argali. The first split separated goat from the *Ovis* species that were grouped as follows: Argali and Urial on one side, and Sardinian mouflon and domestic sheep on the other. In the BI‐tree reconstruction, the Barbary sheep sequence was included within this second clade.

The same pattern of sequences grouping was observed in the Bovinae subfamily where sequences were split into two major clades on the basis of the type of the β‐globin adult gene harbored by the species (Figure 3). Consequently, for example, the HBBA Cattle sequence clustered with HBBA Zebu and Yak rather than with HBBB Cattle. The Water buffalo HBBA and HBBT genes were found basal to the bovine HBBA/HBBB split, followed by the HBB‐X genes.

### 3.3 Molecular Dating of Beta Globin Gene Divergence

To elucidate the evolutionary timeline of the β‐globin genes within Bovidae, we performed a Bayesian molecular dating analysis on the complete nucleotide sequences of the adult β‐globin gene (*HBBA* and *HBBB*). The analysis yielded well‐supported divergence time estimates for key nodes within the phylogeny, with results summarized in Table 5.

**TABLE 5:** Molecular dating estimates for the main splitting events within Bovidae.

Node	MYA	Height 95% HPD
Bovinae/Caprinae HBB	4.16	2.28–6.32
Caprinae HBBA/HBBB	2.55	1.58–3.66
Water buffalo HBBA‐HBBT/other Bovinae	2.3	1.20–3.59
Caprinae HBBA appearance	1.31	0.5–2.22
Caprinae HBBB appearance	1.16	0.41–2.12
Water buffalo HBBA/HBBT	0.64	0.07–1.43
Urial‐HBBA Argali/Sardinian mouflon‐HBBB Sheep	0.56	0.16–1.11
Cattle HBBA/HBBB	0.54	0.19–0.97
HBBB Argali/Bighorn	0.46	0.06–1.04
Cyprian mouflon—HBBK sheep/HBBB Sheep	0.43	0.001–0.32
Urial/HBBA Argali	0.33	0.03–0.73
Sardinian mouflon/HBBA sheep	0.29	0.05–0.65

The deepest divergence within our dataset, that between the bovine and caprine lineages (Bovinae/Caprinae), was estimated to have occurred in the mid‐Miocene, at 4.16 million years ago (MYA) (95% HPD: 2.28–6.32 MYA). Subsequently, the duplication event giving rise to the *HBBA* and *HBBB* paralogs within the Caprinae lineage was dated to the late Pliocene, at 2.55 MYA (95% HPD: 1.58–3.66 MYA).

The divergence between the major caprine genera, *Capra* (goat) and *Ovis* (sheep), was estimated at 1.31 MYA (95% HPD: 0.5–2.22 MYA), placing this event in the early Pleistocene.

Within the genus *Ovis*, our analysis revealed recent and rapid radiation. The split between the Sardinian mouflon and domestic sheep was the most recent, estimated at 0.29 MYA (95% HPD: 0.05–0.65 MYA). The divergence between the Urial and the Argali was also very recent, occurring at 0.33 MYA (95% HPD: 0.03–0.73 MYA), while the split between the Argali and the Bighorn sheep was dated to 0.46 MYA (95% HPD: 0.06–1.04 MYA). The node uniting the Argali‐Bighorn clade with the Cyprian mouflon‐domestic sheep clade was estimated at 1.16 MYA (95% HPD: 0.41–2.12 MYA).

Within the Bovinae, the duplication event leading to the *HBBA* and *HBBB* genes in cattle ( *Bos taurus* ) was estimated to have occurred 0.54 MYA (95% HPD: 0.19–0.97 MYA). Similarly, the divergence between the *HBBA* and *HBBT* paralogs in the water buffalo was dated to 0.64 MYA (95% HPD: 0.07–1.43 MYA). The splitting event between cattle and water buffalo at the *HBBA* locus was estimated at 2.3 MYA (95% HPD: 1.20–3.59 MYA).

## 4 Discussion and Conclusions

In this study, we sequenced the entire adult β‐globin gene from three wild *Ovis* species—Argali, Urial, and Bighorn—with the first two never being sequenced before, and performed a comprehensive comparative phylogenetic analysis to elucidate the evolutionary history of this gene family within Bovidae. Our findings provide robust insights into the phylogenetic relationships and divergence timeline of the β‐globin genes in caprines and bovines.

The analysis of synonymous and non‐synonymous substitutions in the coding portion of the β‐globin gene has highlighted the presence of a purifying selection removing deleterious substitutions and maintaining the sequence highly conserved between domestic and wild sheep. Indeed, the ratio *K*_a_/*K*_s_ quantifying the rate of evolution by stating efficient evolutionary changes in relation to silent background evolutionary changes, and also reflecting the selection pressure on the evolution of organisms, was found to be lower than 1, indicating a negative selection with a reduced rate of fixation of amino acid changes.

The sequence comparison revealed that the greatest divergence was between the paralogous genes *HBBA* and *HBBB*, as expected. More notably, the analysis within the Argali species uncovered the presence of two distinct β‐globin sequences, one clustering with the *HBBA* haplotype and the other with the *HBBB* haplotype of domestic sheep. This finding strongly suggests that the Argali, like domestic sheep, possesses two distinct arrangements of the β‐globin gene cluster. This implies that the genetic polymorphism underlying the two haplotypes is an ancestral trait within the genus *Ovis*, predating the domestication of sheep.

The phylogenetic reconstructions using both BI and ML methods yielded highly congruent and well‐supported trees, with a single discrepancy concerning the placement of the Barbary sheep sequence. The consistent topology confirms a deep evolutionary split within Caprinae into two major clades defined by the type of adult β‐globin gene. The first clade (blue, Figure 3) comprises species carrying the *HBBB/HBBK* genes, including the Bighorn and the *HBBB* Argali sequence, while the second clade (yellow, Figure 3) includes species with the *HBBA* gene, such as the Urial, the other Argali sequence, and the Sardinian mouflon. This pattern of clustering by gene type rather than strictly by species taxonomy was mirrored within the Bovinae subfamily, where sequences grouped according to their paralog (*HBBA*, *HBBB*, or *HBBT*). This consistent phylogenetic signal across two subfamilies underscores the independent evolutionary trajectories of these paralogous genes following duplication, a phenomenon likely driven by concerted evolution or gene conversion within each lineage.

The molecular dating analysis provided a strong temporal framework for these evolutionary events. The estimated divergence between the Bovinae and Caprinae β‐globin gene lineages in the mid‐Miocene (~4.16 MYA; height 95% HPD: 2.28–6.32) does not align with established fossil records for the diversification of the Bovidae family that date back to ~20 MYA (Bibi 2013). This would indicate that such diversification occurred after the separation of the two groups, independently in each lineage. A key finding is the timing of the duplication event that gave rise to the *HBBA* and *HBBB* paralogs within the Caprinae lineage, which we dated to the late Pliocene (~2.55 MYA; height 95% HPD: 1.58–3.66). This estimate is consistent with the result inferred by the analysis of mitochondrial and nuclear markers on the *Ovis* appearance estimated at 2–3 MYA, previously reported (Bibi et al. 2012; Rezaei et al. 2010). However, more recent analyses based on the whole mitogenome sequences dated back the *Ovis* early radiation to 4.6 MYA (Mereu et al. 2025). Such a discrepancy could be explained by assuming that the radiation of the genus *Ovis* event significantly predates the more recent and rapid diversification of the β‐globin gene cluster during the Pleistocene.

Similarly, within Bovinae, the duplication events in Cattle (~0.54 MYA; height 95% HPD: 0.19–0.97) and Water buffalo (~0.64 MYA; height 95% HPD: 0.07–1.43) are also recent, occurring long after the split of the bovine ancestral β‐globin gene (~2.3 MYA). This indicates that the *HBBA/HBBB* paralogs in Cattle and the *HBBA/HBBT* paralogs in Water buffalo are the result of independent, lineage‐specific duplication events, further highlighting the dynamic evolution of the β‐globin cluster in ruminants (Dutta et al. 2020).

In conclusion, even taking into account the constrained sampling, our integrated analysis of sequence variation, phylogeny, and molecular dating demonstrates that the evolution of the β‐globin gene family in Bovidae is characterized by ancient paralogous diversification followed by recent conspecific radiation. The confirmation of two β‐globin haplotypes in the wild Argali provides important context for understanding the genetic basis of Hb variation in domestic sheep. The well‐resolved phylogeny and specific divergence time estimates presented here offer a valuable resource for future studies on mammalian molecular evolution and the evolutionary history of the genus *Ovis*.

## Author Contributions

**Paolo Mereu:** conceptualization (lead), formal analysis (equal), investigation (equal), writing – original draft (lead), writing – review and editing (equal). **Chiara Multineddu:** conceptualization (equal), investigation (lead), writing – review and editing (equal). **Daria Sanna:** formal analysis (equal), investigation (equal), writing – review and editing (equal). **Marco Zedda:** formal analysis (equal), supervision (equal), writing – review and editing (equal). **Antonio J. Lepedda:** formal analysis (equal), writing – review and editing (equal). **Giovanni G. Leoni:** formal analysis (equal), supervision (lead), writing – review and editing (equal). **Monica Pirastru:** formal analysis (equal), investigation (equal), writing – review and editing (equal).

## Conflicts of Interest

The authors declare no conflicts of interest.

## Supporting information


**Table S1:** Estimates of evolutionary divergence between *Ovis* species sequences.

## Data Availability

The sequences produced in the present study have been deposited in GenBank under the accession numbers PX441602, PX441603, PX441604, and PX441605.

## References

[ece373031-bib-0001] Bibi, F. 2013. “A Multi‐Calibrated Mitochondrial Phylogeny of Extant Bovidae (Artiodactyla, Ruminantia) and the Importance of the Fossil Record to Systematics.” BMC Evolutionary Biology 13: 166. 10.1186/1471-2148-13-166. 23927069 PMC3751017

[ece373031-bib-0002] Bibi, F. , E. Vrba , and F. Fack . 2012. “A New African Fossil Caprin and a Combined Molecular and Morphological Bayesian Phylogenetic Analysis of Caprini (Mammalia: Bovidae).” Journal of Evolutionary Biology 25: 1843–1854. 10.1111/j.1420-9101.2012.02572.x. 22816969

[ece373031-bib-0003] Chen, Z.‐H. , Y.‐X. Xu , X.‐L. Xie , et al. 2021. “Whole‐Genome Sequence Analysis Unveils Different Origins of European and Asiatic Mouflon and Domestication‐Related Genes in Sheep.” Communications Biology 4: 1307. 10.1038/s42003-021-02817-4. 34795381 PMC8602413

[ece373031-bib-0004] Dotsev, A. , O. Koshkina , V. Kharzinova , et al. 2023. “Genome‐Wide Insights Into Intraspecific Taxonomy and Genetic Diversity of Argali (*Ovis ammon*).” Diversity 15: 627. 10.3390/d15050627.

[ece373031-bib-0005] Dutta, P. , A. Talenti , R. Young , et al. 2020. “Whole Genome Analysis of Water Buffalo and Global Cattle Breeds Highlights Convergent Signatures of Domestication.” Nature Communications 11: 4739. 10.1038/s41467-020-18550-1. PMC7505982 32958756

[ece373031-bib-0006] Hardison, R. C. 2012. “Evolution of Hemoglobin and Its Genes.” Cold Spring Harbor Perspectives in Medicine 2: a011627. 10.1101/cshperspect.a011627. 23209182 PMC3543078

[ece373031-bib-0007] Jiang, Y. , X. Wang , J. W. Kijas , and B. P. Dalrymple . 2015. “Beta‐Globin Gene Evolution in the Ruminants: Evidence for an Ancient Origin of Sheep Haplotype B.” Animal Genetics 46: 506–514. 10.1111/age.12318. 26096044

[ece373031-bib-0008] Kumar, S. , G. Stecher , M. Suleski , and S. B. Hedges . 2017. “TimeTree: A Resource for Timelines, Timetrees, and Divergence Times.” Molecular Biology and Evolution 34: 1812–1819. 10.1093/molbev/msx116. 28387841

[ece373031-bib-0009] Larkin, M. A. , G. Blackshields , N. P. Brown , et al. 2007. “Clustal W and Clustal X Version 2.0.” Bioinformatics 23: 2947–2948. 10.1093/bioinformatics/btm404. 17846036

[ece373031-bib-0010] Lv, F.‐H. , D.‐F. Wang , S.‐Y. Zhao , et al. 2024. “Deep Ancestral Introgressions Between Ovine Species Shape Sheep Genomes via Argali‐Mediated Gene Flow.” Molecular Biology and Evolution 41: msae212. 10.1093/molbev/msae212. 39404100 PMC11542629

[ece373031-bib-0011] MacEachern, S. , J. McEwan , and M. Goddard . 2009. “Phylogenetic Reconstruction and the Identification of Ancient Polymorphism in the Bovini Tribe (Bovidae, Bovinae).” BMC Genomics 10: 177. 10.1186/1471-2164-10-177. 19393045 PMC2694835

[ece373031-bib-0012] Manca, L. , M. Pirastru , P. Mereu , et al. 2006. “Barbary Sheep (*Ammotragus lervia*): The Structure of the Adult Beta‐Globin Gene and the Functional Properties of Its Hemoglobin.” Comparative Biochemistry and Physiology. Part B, Biochemistry & Molecular Biology 145: 214–219. 10.1016/j.cbpb.2006.07.010. 16962804

[ece373031-bib-0013] Mereu, P. , M. Pirastru , P. Morell Miranda , et al. 2025. “Revised Phylogeny of Mouflon Based on Expanded Sampling of Mitogenomes.” PLoS One 20: e0323354. 10.1371/journal.pone.0323354. 40367058 PMC12077669

[ece373031-bib-0014] Mereu, P. , M. Pirastru , D. Sanna , G. Bassu , S. Naitana , and G. G. Leoni . 2024. “Phenotype Transition From Wild Mouflon to Domestic Sheep.” Genetics Selection Evolution 56: 1. 10.1186/s12711-023-00871-6. PMC10763062 38166592

[ece373031-bib-0026] Nei, M. , and T. Gojobori . 1986. “Simple methods for estimating the numbers of synonymous and nonsynonymous nucleotide substitutions.” Molecular Biology and Evolution 3, no. 5: 418–426. 10.1093/oxfordjournals.molbev.a040410. 3444411

[ece373031-bib-0015] Pirastru, M. , L. Manca , and B. Masala . 2003. “Characterization of Four Novel Variants of Goat βA‐Globin Gene.” Biochemical Genetics 41: 209–217. 14587664 10.1023/a:1025547517169

[ece373031-bib-0016] Pirastru, M. , C. Multineddu , P. Mereu , et al. 2009. “The Sequence and Phylogenesis of the α‐Globin Genes of Barbary Sheep ( *Ammotragus lervia* ), Goat ( *Capra hircus* ), European Mouflon ( *Ovis aries musimon* ) and Cyprus Mouflon ( *Ovis aries ophion* ).” Comparative Biochemistry and Physiology Part D: Genomics and Proteomics 4: 168–173. 10.1016/j.cbd.2009.02.002. 20403763

[ece373031-bib-0017] Rangan, A. , M. S. Hein , W. G. Jenkinson , et al. 2021. “Improved Characterization of Complex β‐Globin Gene Cluster Structural Variants Using Long‐Read Sequencing.” Journal of Molecular Diagnostics 23: 1732–1740. 10.1016/j.jmoldx.2021.08.013. 34839893

[ece373031-bib-0018] Rezaei, H. R. , S. Naderi , I. C. Chintauan‐Marquier , et al. 2010. “Evolution and Taxonomy of the Wild Species of the Genus *Ovis* (Mammalia, Artiodactyla, Bovidae).” Molecular Phylogenetics and Evolution 54: 315–326. 10.1016/j.ympev.2009.10.037. 19897045

[ece373031-bib-0019] Ronquist, F. , M. Teslenko , P. Van Der Mark , et al. 2012. “MrBayes 3.2: Efficient Bayesian Phylogenetic Inference and Model Choice Across a Large Model Space.” Systematic Biology 61: 539–542. 10.1093/sysbio/sys029. 22357727 PMC3329765

[ece373031-bib-0020] Rozas, J. , A. Ferrer‐Mata , J. C. Sánchez‐DelBarrio , et al. 2017. “DnaSP 6: DNA Sequence Polymorphism Analysis of Large Data Sets.” Molecular Biology and Evolution 34: 3299–3302. 10.1093/molbev/msx248. 29029172

[ece373031-bib-0021] Stamatakis, A. 2014. “RAxML Version 8: A Tool for Phylogenetic Analysis and Post‐Analysis of Large Phylogenies.” Bioinformatics 30: 1312–1313. 10.1093/bioinformatics/btu033. 24451623 PMC3998144

[ece373031-bib-0022] Storz, J. F. 2016. “Gene Duplication and Evolutionary Innovations in Hemoglobin‐Oxygen Transport.” Physiology 31: 223–232. 10.1152/physiol.00060.2015. 27053736 PMC5005275

[ece373031-bib-0023] Storz, J. F. 2018. “Evolution of the Vertebrate Globin Gene Family.” In Hemoglobin: Insights Into Protein Structure, Function, and Evolution. Oxford University Press.

[ece373031-bib-0024] Suchard, M. A. , P. Lemey , G. Baele , D. L. Ayres , A. J. Drummond , and A. Rambaut . 2018. “Bayesian Phylogenetic and Phylodynamic Data Integration Using BEAST 1.10.” Virus Evolution 4: vey016. 10.1093/ve/vey016. 29942656 PMC6007674

[ece373031-bib-0025] Tamura, K. , G. Stecher , and S. Kumar . 2021. “MEGA11: Molecular Evolutionary Genetics Analysis Version 11.” Molecular Biology and Evolution 38: 3022–3027. 10.1093/molbev/msab120. 33892491 PMC8233496

