# Supplementary material for: Evolutionary History of the Adult β‐Globin Gene in Wild Sheep: First Sequences From the Argali (Ovis ammon) and the Urial (Ovis vignei)
Source: Ecol Evol. 2026 Feb 23;16(2):e73031. doi: 10.1002/ece3.73031 (PMC12929664; doi:10.1002/ece3.73031)
Supplement: Supplementary file 1 — Table S1: Estimates of evolutionary divergence between Ovis species sequences. [file ECE3-16-e73031-s001.docx]

Table S1. Estimates of evolutionary divergence between *Ovis* species sequences

|  | HBBK Sheep | HBBB Argali | HBBA Argali | Bighorn | Urial | HBBB Sheep (Sar) | HBBA Sheep (Sar) | Sardinian mouflon | Cyprian mouflon | HBBB Sheep (G&L) | HBBB Sheep (Bra) |
| --- | --- | --- | --- | --- | --- | --- | --- | --- | --- | --- | --- |
| HBBK Sheep |  | 0,00303 | 0,00417 | 0,00305 | 0,00387 | 0,00138 | 0,00418 | 0,00418 | 0,00071 | 0,00153 | 0,00138 |
| HBBB Argali | 0,01331 |  | 0,00315 | 0,00170 | 0,00308 | 0,00289 | 0,00349 | 0,00332 | 0,00294 | 0,00298 | 0,00289 |
| HBBA Argali | 0,02410 | 0,01334 |  | 0,00312 | 0,00141 | 0,00405 | 0,00210 | 0,00186 | 0,00409 | 0,00412 | 0,00405 |
| Bighorn | 0,01331 | 0,00417 | 0,01334 |  | 0,00305 | 0,00287 | 0,00348 | 0,00330 | 0,00295 | 0,00296 | 0,00287 |
| Urial | 0,02118 | 0,01334 | 0,00277 | 0,01334 |  | 0,00374 | 0,00182 | 0,00157 | 0,00378 | 0,00382 | 0,00374 |
| HBBB Sheep (Sar) | 0,00277 | 0,01190 | 0,02266 | 0,01190 | 0,01976 |  | 0,00406 | 0,00408 | 0,00119 | 0,00068 | 0,00000 |
| HBBA Sheep (Sar) | 0,02481 | 0,01692 | 0,00627 | 0,01692 | 0,00487 | 0,02338 |  | 0,00169 | 0,00410 | 0,00413 | 0,00406 |
| Sardinian mouflon | 0,02481 | 0,01548 | 0,00487 | 0,01548 | 0,00347 | 0,02338 | 0,00416 |  | 0,00411 | 0,00415 | 0,00408 |
| Cyprian mouflon | 0,00069 | 0,01259 | 0,02337 | 0,01259 | 0,02046 | 0,00208 | 0,02408 | 0,02408 |  | 0,00136 | 0,00119 |
| HBBB Sheep (G&L) | 0,00347 | 0,01260 | 0,02338 | 0,01260 | 0,02047 | 0,00069 | 0,02410 | 0,02410 | 0,00277 |  | 0,00068 |
| HBBB Sheep (Bra) | 0,00277 | 0,01190 | 0,02266 | 0,01190 | 0,01976 | 0,00000 | 0,02338 | 0,02338 | 0,00208 | 0,00069 |  |

The number of base substitutions per site from between sequences are shown. Standard error estimate(s) are shown above the diagonal. Analyses were conducted using the Tamura 3-parameter model. The rate variation among sites was modeled with a gamma distribution (shape parameter = 2.78). This analysis involved 12 nucleotide sequences. Codon positions included were 1st+2nd+3rd+Noncoding. All positions containing gaps and missing data were eliminated (complete deletion option). There were a total of 1447 positions in the final dataset.
